# A Stable Delivery System for *Meretrix meretrix* Derived Immunomodulatory Peptide (QLNWD): Fabrication and Characterization of Glycosylated Protein Nanoparticle

**DOI:** 10.3390/md23100385

**Published:** 2025-09-27

**Authors:** Wanyi Wu, Zhixuan Wu, Jiamin Cai, Wenhong Cao, Haisheng Lin, Jialong Gao, Xiuping Fan, Huina Zheng, Xiaoming Qin

**Affiliations:** 1 National Research and Development Branch Center of Shellfish Processing (Zhanjiang), Guangdong Provincial Key Laboratory of Aquatic Products Processing and Safety, College of Food Science and Technology, Guangdong Ocean University, Zhanjiang 524088, China; wanyi6265@163.com (W.W.); wzx8201003@163.com (Z.W.); cjm170022@163.com (J.C.); cwenhong@gdou.edu.cn (W.C.); haishenglin@163.com (H.L.); gaojl@gdou.edu.cn (J.G.); fanxp08@163.com (X.F.); 2 Shenzhen Institute, Guangdong Ocean University, Shenzhen 518108, China; 3 Southern Marine Science and Engineering Guangdong Laboratory (Zhanjiang), Zhanjiang 524000, China

**Keywords:** marine immunomodulatory peptide, *Meretrix meretrix*, Maillard reaction, self-assembly, glycosylated protein nanoparticle, stabilization

## Abstract

In this study, nanoparticles prepared by the heat-induced self-assembly of bovine serum albumin-dextran conjugates (BSA-DX) were utilized as an effective delivery system for the immunoregulatory peptide Gln-Leu-Asn-Trp-Asp (QLNWD) from *Meretrix meretrix*. The effects of dry-heating duration on the fabrication and characteristics of glycoprotein nanoparticles (GBA NPs) were investigated. Stable GBA NPs (110.84 nm) were obtained after 9 h of dry-heating. Depending on the addition sequence of QLNWD, the QLNWD-loaded nanoparticles were categorized into two types: pre-loading and post-loading. The two strategies were evaluated based on physicochemical characterization, colloidal stability, and RAW264.7 macrophage uptake. Results showed that upon QLNWD incorporation, both pre-loading NPs and post-loading NPs exhibited spherical morphology, with particle sizes decreasing to 105.51 nm and 94.27 nm, respectively. The encapsulation efficiency of pre-loading NPs for QLNWD was higher (87.74%), and the co-localization ability between post-loading NPs and QLNWD was stronger (Pearson’s correlation coefficient = 0.95). In vitro simulated gastrointestinal digestion experiments showed that QLNWD bioaccessibility increased to 47.5% and 42.7% for pre-loaded and post-loaded NPs, respectively. Compared to free QLNWD, NP encapsulation significantly enhanced the uptake of QLNWD by macrophages. Thus, GBA NPs, particularly those prepared by the pre-loading method, are considered promising delivery systems for marine bioactive peptides.

## 1. Introduction

Marine organisms are a rich source of bioactive peptides with unique structures and diverse biological activities [1]. These peptides exhibit high specificity, low toxicity, and good biocompatibility, making them promising candidates for innovative therapeutics and various applications [2]. By interacting with biofilms, modulating immune responses, and inhibiting or activating specific enzymes and receptors, marine peptides demonstrate antibacterial, immunomodulatory, anticancer, and other therapeutic effects. In particular, marine immunomodulatory peptides can enhance the body’s defense mechanisms by regulating both innate and adaptive immunity [3]. In a previous study, Zhang et al. [4] employed a cyclophosphamide (Cy)-induced immunosuppressive mouse model to evaluate the immunomodulatory activity of the active peptide Gln-Leu-Asn-Trp-Asp (QLNWD), which was derived from the visceral hydrolysate of *Meretrix meretrix*. The results demonstrated that intraperitoneal injection of QLNWD alleviated Cy-induced weight loss and atrophy of the thymus and spleen in mice. Furthermore, it promoted the proliferation and differentiation of T-lymphocytes in immune tissues and enhanced the production of immunoglobulin G and hemolysin in serum, thereby ameliorating Cy-induced immunosuppression. It is noteworthy that marine peptide drugs still face challenges in clinical applications, including poor stability, low bioavailability, and insufficient targeting [5]. Oral administration is the most convenient and widely accepted route for patients, and the efficacy of peptide drugs largely depends on their ability to maintain structural integrity before reaching target cells. However, the complex physiological environment of the gastrointestinal tract and the low permeability of the intestinal epithelium pose major barriers to oral delivery [6].

In recent years, the development of nano-delivery systems has provided new strategies to enhance the delivery efficiency and bioavailability of peptide drugs, particularly peptide-based self-assembled nanosystems, which offer notable advantages including simplicity, programmability, and excellent biocompatibility [7]. Cheng et al. [8] designed a multifunctional peptide-assembled nanoparticle with dual responsiveness and intrinsic therapeutic potential, enabling precise control of drug release to enhance bioavailability and reduce adverse reactions in tumor immunotherapy. In addition, the hydrophilic peptide moiety enables synergistic delivery with hydrophobic drugs. A peptide-based nanogel loaded with dexamethasone exhibits up to 90 days of storage stability, shows no hemotoxicity, and is rapidly internalized by leukemia cells [9]. The design of nanosystems targeting marine peptides can enhance their targeting ability and stability.

Encapsulation technology using biopolymer carriers, such as proteins and polysaccharides, has proven to be an effective strategy for protecting sensitive bioactive compounds and enhancing their stability under physiological conditions, particularly by significantly reducing drug toxicity [10]. Nanostructures with favorable biocompatibility minimize direct exposure of peptide drugs to the external environment, while the modifiability of carriers enables targeted delivery [11]. The formation of covalent conjugates between proteins and high-molecular-weight nonionic polysaccharides via the Maillard reaction can significantly enhance the solubility, emulsifying activity, and stability of proteins by leveraging the steric hindrance effect of polysaccharide chains [12]. Glycated proteins, as amphiphilic copolymers, can form nanogel particles with a hydrophobic protein core and a hydrophilic polysaccharide shell through heat-induced self-assembly. This method, which combines the Maillard reaction and self-assembly, is characterized by simple operation, mild reaction conditions, and excellent environmental compatibility [13].

Bovine serum albumin (BSA) is a protein with abundant availability and low cost, exhibiting excellent biocompatibility and biodegradability. Upon thermal denaturation and aggregation, it can form a three-dimensional gel network structure, which contributes to its widespread application in the design of nanocarriers [14]. Dextran (DX) is a highly branched, water-soluble polysaccharide that not only enables targeting of macrophages in atherosclerotic plaques but also serves as an excellent hydrophilic component in the construction of BSA conjugates [15]. Their application potential in marine peptide delivery has not yet been reported. There is still a lack of systematic comparative analysis on the advantages and limitations of different peptide loading sequences on nano-delivery systems, including effects on encapsulation efficiency, molecular stability, and release profile.

The purpose of this study is to construct a novel and biodegradable peptide-based nano-delivery system for the immunomodulatory pentapeptide QLNWD, aiming to enhance its stability and achieve controlled release. Glycoprotein nanoparticles (GBA NPs) were prepared via the thermal gelation method using bovine serum albumin-dextran conjugate (BSA-DX), synthesized by the dry Maillard reaction, as the matrix, and the potential for QLNWD delivery was evaluated. By comparing two encapsulation strategies (loading before gelation and adsorption after gelation) in terms of their effects on key nanoparticle properties, including physicochemical characteristics, colloidal stability, and macrophage-targeting ability, differences in the behavior of peptide molecules during system self-assembly were analyzed. This work is expected to provide an optimization strategy for efficient marine peptide delivery.

## 2. Results

### 2.1. Characterization of the BSA-DX Conjugates

**Figure 1:**
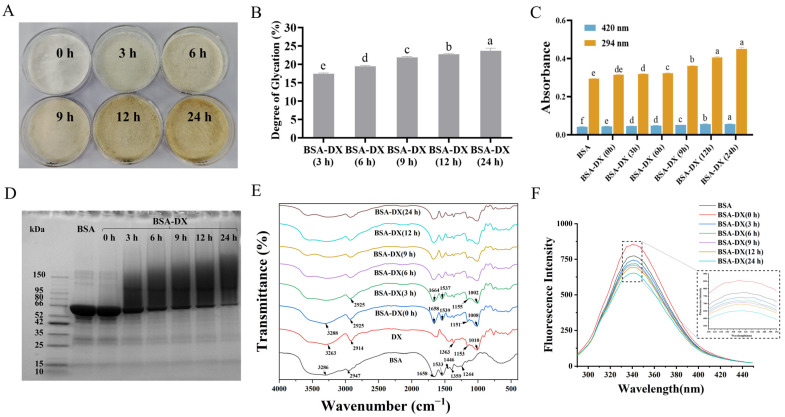
(**A**) Bovine serum albumin-dextran conjugates (BSA-DX) at 0, 3, 6, 9, 12, and 24 h. (**B**) Degree of glucosylation. (**C**) Browning Index. (**D**) Sodium dodecyl sulfate-polyacrylamide gel electrophoresis (SDS-PAGE). (**E**) Fourier transform-infrared spectroscopy (FT-IR). (**F**) Fluorescence spectra. Data were represented as mean ± SD (*n* = 3). Different letters indicated the significant difference at *p* < 0.05.

The BSA-DX conjugate was synthesized through a covalent linkage between the carboxyl group at the reducing end of DX and the amino group of BSA, facilitated by the Maillard reaction. As shown in the sample diagram of Figure 1A, the prolonged Maillard reaction time leads to a gradual darkening of the conjugate’s color. The degree of glycosylation (DG) of BSA-DX conjugates increased as the reaction time extended from 0 to 24 h. The DG value reached (21.90 ± 0.24)% at 9 h, and the maximum value of (23.67 ± 0.71)% was observed at 24 h, indicating continuous conjugate formation during the dry-heat treatment (Figure 1B). Melanoidin content after grafting was measured at 420 nm, while 294 nm absorbance indicated early Maillard reaction intermediates. To improve protein functionality and stability, the Maillard grafting is usually limited to the early reaction stage to avoid advanced product formation [16]. The much higher A_294 nm_ than A_420 nm_ values during 3–24 h of dry-heat treatment confirm that grafting mainly occurs in the initial Maillard reaction phase (Figure 1C). The sodium dodecyl sulfate-polyacrylamide gel electrophoresis (SDS-PAGE) results are shown in Figure 1D. At 0 h, the band aligned with BSA, indicating no successful coupling between BSA and DX. As reaction time increased, the BSA band weakened, and a dense, continuous band emerged in the high molecular mass region, gradually intensifying with time. This indicates successful covalent coupling of BSA and DX to form a polymer complex, and the dynamic change is consistent with the DG results.

In Figure 1E, the characteristic absorption peaks of natural BSA are observed at: amide I band 1658 cm^−1^ (C=O stretching vibration), amide II band 1533 cm^−1^ (N-H bending vibration), and amide III band 1244 cm^−1^ (C-N stretching and NH deformation vibrations) [17]. Compared with the physical mixture BSA-DX (0 h), as the Maillard reaction time increases, changes in the amide bands of the protein become evident, particularly a gradual decrease in the intensity of the amide II band. This indicates substantial consumption of the protein’s free amino groups (-NH_2_) during the reaction, which participate in the formation of covalent bonds. The intensity of the broad band at 3200–3600 cm^−1^ (attributed to overlapping O-H and N-H stretching vibrations) gradually increases, primarily due to the introduction of polysaccharide molecules rich in O-H groups. The sugar band of DX appears in the range of 1180–953 cm^−1^, mainly arising from C-O-C, C-O, and C-H vibrations. The absorption intensity of BSA-DX in this region is significantly higher than that of pure BSA but lower than that of the BSA-DX (0 h), confirming the occurrence of glycosylation in BSA-DX. The maximum fluorescence intensity (Figure 1F) of BSA-DX conjugates gradually decreased compared to BSA, along with a slight red shift. In short, the above results confirm that the BSA-DX conjugates were formed via the Maillard reaction.

### 2.2. Characteristics of GBA NPs

**Figure 2:**
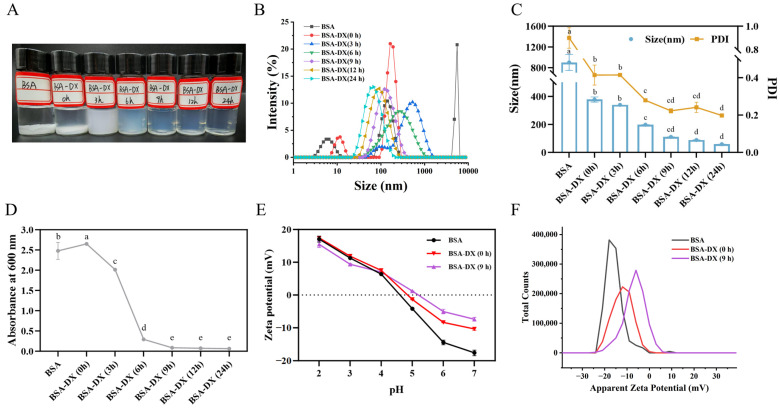
Protein nanoparticle (**A**), size distribution (**B**), particle size (**C**), and turbidity (**D**) were prepared with native BSA, BSA-DX conjugate glycosylation for 0, 3, 6, 12, and 24 h, respectively. Effects of pH (2.0–7.0) on zeta-potential (**E**) and its distribution profiles (at pH 7.0) (**F**) of protein nanoparticle prepared from native BSA, BSA-DX Maillard conjugates at 0 and 9 h. Different letters indicated the significant difference at *p* < 0.05.

Heating BSA at 80 °C for 10 min leads to the formation of a rigid BSA network [18]. Moreover, heating BSA above its denaturation temperature range of 60–72 °C results in complete structural unfolding, thereby facilitating the formation of nanogel particles. The nanogel particle samples formed by BSA and BSA-DX conjugates under different dry-heating durations are presented in Figure 2A. Pure BSA underwent aggregation after heating at pH 5.2. The BSA-DX conjugate was subjected to dry-heating treatment for 3 h, resulting in the formation of bright blue nanogels.

According to the dynamic light scattering (DLS) results (Figure 2B), a single peak and narrow particle size intensity distribution were observed after 9 h of reaction time. As shown in Figure 2C, the particle size and PDI of the NPs decrease significantly with increasing dry-heating time, indicating that a higher degree of grafting provides more effective stabilization through enhanced steric hindrance of the NPs. At 6 h, the particle size of the NPs reached (197.46 ± 0.73) nm, and NPs with uniform dispersion (PDI < 0.4) were formed. The smallest particle size of (58.64 ± 0.53) nm was observed at 24 h. The PDI gradually decreased with increasing reaction time and eventually stabilized at a value close to 0.2. The turbidity values of the samples (Figure 2D) are consistent with the DLS data, showing no significant differences among the samples after 9 h. As shown in the zeta potential results from Figure 2E, when the pH is above the isoelectric point, the absolute zeta potential value of the NPs formed after 9 h of reaction is lower than that of native BSA. In particular, at pH 7, the potential distribution maps are shown in Figure 2F. BSA-DX (9 h) exhibits a single sharp peak, with a significantly narrower peak width compared to that of BSA-DX (0 h). This shift toward zero potential is consistent with the decreasing trend in the average Zeta potential observed in Figure 2E. Therefore, in subsequent experiments, GBA NPs obtained by dry-heating at 60 °C for 9 h were selected for the encapsulation of the immunoregulatory peptide QLNWD.

### 2.3. Characterization of GBA NPs Loading QLNWD

#### 2.3.1. Particulate and Morphological Characteristics

Depending on whether QLNWD was added before or after nanoparticle formation, the preparation methods were classified as pre-loading and post-loading for comparative analysis. The effects of these two encapsulation approaches on the particle size and zeta potential of the NPs were evaluated, as presented in Table 1. The average diameter of GBA NPs was 110.84 nm, with a PDI of 0.223 and a zeta potential of −7.38 mV. Following QLNWD encapsulation, the particle sizes of pre-loading NPs and post-loading NPs decreased to 105.51 nm and 94.27 nm, respectively. In addition, the absolute zeta potential values of both pre-loading NPs and post-loading NPs increased by approximately 1 mV during the encapsulation process.

**Figure 3:**
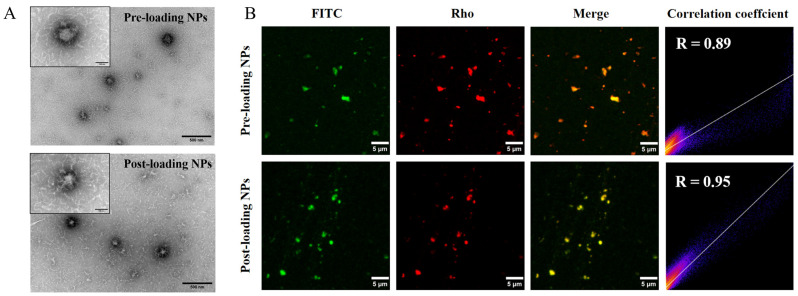
(**A**) Transmission electron microscopy (TEM) images of nanoparticles before loading and after loading. Insets display high-magnification views. Scale bars: 500 nm for main images and 100 nm for insets. (**B**) Fluorescence co-localization images of FITC-labeled Gln-Leu-Asn-Trp-Asp (QLNWD) (green) and Rho-labeled NPs (red). Scale bar: 5 μm.

The freshly prepared pre-loading NPs and post-loading NPs were morphologically analyzed using transmission electron microscopy (TEM) (Figure 3A). Both types of NPs exhibit an approximately spherical morphology and a uniform size distribution. The particle size of pre-loading NPs is slightly larger than that of post-loading NPs, a finding that is consistent with the trend observed in DLS measurements. The co-localization of NPs and QLNWD was further assessed through fluorescence localization. As shown in Figure 3B, the red fluorescence of the nanogel particles overlaps with the green fluorescence of QLNWD, indicating strong co-localization between the NPs and QLNWD. Pearson’s correlation coefficient (R) for post-loading NPs was 0.95, higher than that of pre-loading NPs (0.89).

#### 2.3.2. Physicochemical Characterization

**Figure 4:**
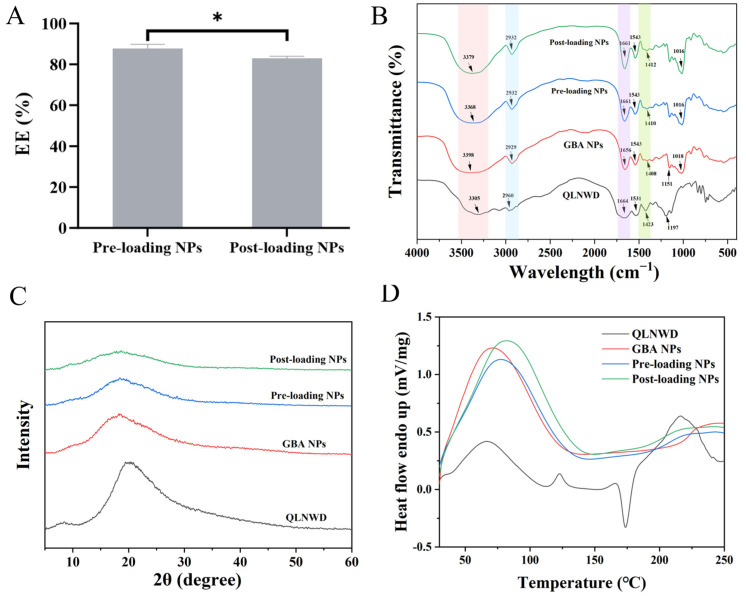
(**A**) Encapsulation efficiency (EE) of pre-loading NPs and post-loading NPs. FTIR spectra (**B**), X-ray diffraction (XRD) patterns (**C**), and differential scanning calorimetry (DSC) spectra (**D**) of QLNWD, GBA NPs, pre-loading NPs, and post-loading NPs. Asterisks indicate significant differences: (*) *p* < 0.05.

Although the two QLNWD-loaded NPs are composed of the same material, they exhibit different EE% of QLNWD, as shown in Figure 4A. Specifically, the pre-loading NPs demonstrate an EE of (87.74 ± 2.02)%, whereas the post-loading NPs show a slightly lower EE of (82.95 ± 1.03)%. Fourier transform-infrared spectroscopy (FT-IR) was employed to investigate the interaction between QLNWD and NPs (Figure 4B). In the spectra of QLNWD and GBA NPs, the characteristic peaks of the amide I band were observed at 1664 cm^−1^ and 1656 cm^−1^, respectively, while the amide II band peaks appeared at 1423 cm^−1^ and 1408 cm^−1^, respectively. Following the encapsulation of QLNWD, both the amide I and amide II bands in the pre-loading NPs and post-loading NPs exhibited a red shift, which may be attributed to hydrophobic and hydrogen bonding interactions between the NPs and QLNWD [19]. At the same time, the NPs exhibited a characteristic broad band at 3398 cm^−1^, corresponding to the O-H stretching vibration of the dextran moiety. The O-H stretching vibrations of the pre-loading NPs and post-loading NPs showed blue shifts to 3368 cm^−1^ and 3379 cm^−1^, respectively, indicating the involvement of hydrogen bonding. Interestingly, the interaction between the two nanocarriers and QLNWD remains unaffected by the sequence of material addition.

The alteration in the crystalline state of NPs following QLNWD encapsulation was analyzed using X-ray diffraction (XRD). As illustrated in Figure 4C, QLNWD exhibited a distinct diffraction peak at 20.5°, whereas GBA NPs displayed a broad amorphous band at 18.2°. After subtracting the background signal from GBA NPs (963 intensity units at 20.5°), the net intensity attributable to QLNWD was negative (Pre: −282; Post: −476), indicating successful encapsulation of the peptide within the NPs and its amorphous state. The thermal stability of QLNWD and QLNWD-loaded NPs during heating was evaluated using differential scanning calorimetry (DSC). As shown in Figure 4D, QLNWD exhibited endothermic peaks at 66.7 °C, 117.0 °C, and 216.3 °C, along with a cold-crystallization exotherm at 173.8 °C (ΔH = −20.57 J/g). This exothermic peak was completely absent in both NPs. The peak melting temperature (Tm) of GBA NPs was recorded at 70.3 °C, and the broader endothermic peak suggests that heating led to the exposure of hydrophobic regions within the protein structure [17]. Upon the addition of QLNWD to the NPs, the dehydration temperatures of the pre-loading NPs and post-loading NPs increased to 76.8 °C and 80.7 °C, respectively, which supports the findings from the previous XRD analysis. These results indicate a reduction in the crystallinity of QLNWD within the NPs, with the post-loading NPs demonstrating the highest thermal stability.

### 2.4. Evaluation of Physical Stability

**Figure 5:**
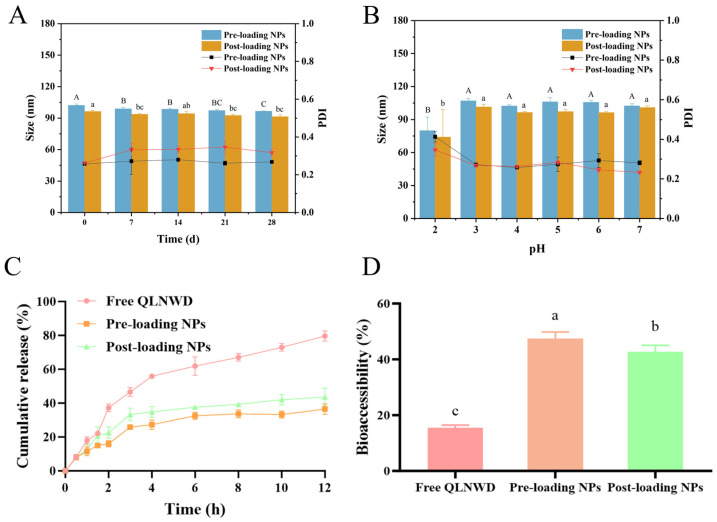
The stability of pre-loaded NPs and post-loaded NPs, and the sustained release property of QLNWD. (**A**) Storage stability at 4 °C, (**B**) pH stability, (**C**) release behaviors during simulated gastric and intestinal digestion (enzyme-free), (**D**) bioaccessibility of QLNWD (enzyme-containing). Different letters indicated the significant difference at *p* < 0.05.

The effects of storage duration and pH on the stability of pre-loading NPs and post-loading NPs were further investigated. After 28 days of storage at 4 °C, the particle size and PDI of both pre-loading NPs and post-loading NPs were measured at specified time intervals (Figure 5A). With increasing storage duration, the particle size of the nanoparticles gradually decreased. Notably, the PDI of post-loading NPs exceeded 0.3 after seven days of storage, whereas the PDI of pre-loading NPs remained consistently below 0.3. This suggests that pre-loading NPs exhibit enhanced storage stability, indicating their initial thermodynamic equilibrium upon the formation of nanogel particles [20]. The particle size and PDI of pre-loading NPs and post-loading NPs under varying pH conditions are presented in Figure 5B. Both types of NPs maintained a comparable average size across the pH range of 3.0 to 7.0. These findings demonstrate that the QLNWD-loaded NPs exhibited stability during 28 days of storage at 4 °C, as well as under slightly acidic and neutral pH conditions.

### 2.5. In Vitro Release Profile and Bioaccessibility of QLNWD

Through the dialysis bag method, SGF and SIF without enzymes were employed to investigate the sustained release profiles of pre-loading NPs and post-loading NPs for QLNWD. This setup is designed to elucidate the intrinsic controlled-release properties of the nanoparticles by eliminating the confounding effects of enzymatic degradation. As shown in Figure 5C, during the initial 2 h, pre-loading NPs and post-loading NPs released 14.9% and 22.5% of QLNWD, respectively, which were significantly lower than the 38.1% release observed for free QLNWD. This indicates that the nanoparticles effectively retarded the release of QLNWD in SGF. Subsequently, the environmental pH returned to neutral conditions. When the simulated digestion reached 12 h, the cumulative release of QLNWD from pre-loading NPs and post-loading NPs peaked at 37.8% and 40.6%, respectively. This indicates that the release process was prolonged compared to free QLNWD, which had reached 79.1% release at the same time point. The release rate of QLNWD from pre-loading NPs was significantly slower than that from post-loading NPs.

The bioaccessibility of QLNWD was evaluated in SGF/SIF containing digestive enzymes (pepsin and pancreatin), reflecting the system’s resistance to enzymatic degradation. As shown in Figure 5D, the bioaccessibility of free QLNWD during the gastric digestion phase was only 15.5%. In contrast, the bioaccessibility of pre-loading NPs and post-loading NPs increased to 47.5% and 42.7%, respectively, demonstrating that the delivery system significantly enhances bioaccessibility. In summary, both pre-loading NPs and post-loading NPs exhibit favorable gastrointestinal digestion stability and sustained release properties, with pre-loading NPs showing particularly strong potential. These characteristics provide evidence that they can serve as effective oral carriers for improving the bioaccessibility of bioactive peptides.

### 2.6. Cytotoxicity Assessment

**Figure 6:**
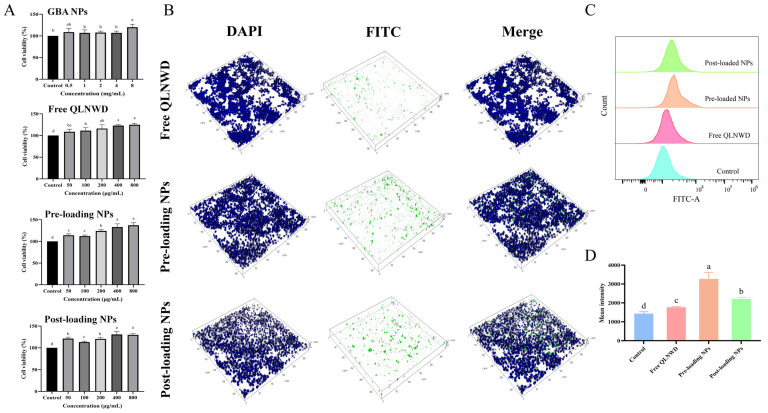
(**A**) Effect of GBA NPs, free QLNWD, pre-loading NPs, and post-loading NPs on the viability of RAW264.7 cells. (**B**) The 3D confocal laser scanning microscope (CLSM) images of the RAW264.7 cellular uptake of free QLNWD and QLNWD-loaded nanoparticles. Nuclei were stained with DAPI (blue). QLNWD was labeled with FITC (green). The flow cytometric profiles (**C**) and mean intensity (**D**) of NPs uptake by RAW264.7 cells. Different letters indicated the significant difference at *p* < 0.05.

The biocompatibility of QLNWD, GBA NPs, pre-loading NPs, and post-loading NPs was evaluated using the Cell Counting Kit-8 (CCK-8) assay. As shown in Figure 6A, within the concentration range of 0.5–8 mg/mL, the GBA NPs group exhibited no cytotoxic effects on RAW264.7 cells after 24 h of incubation. The cell viability of the pre-loading NPs and post-loading NPs increased in a dose-dependent manner following 24-h exposure. When the final concentration of QLNWD reached 800 μg/mL, the cell proliferation rates for pre-loading NPs and post-loading NPs were (137.23 ± 5.92)% and (129.85 ± 2.74)%, respectively, which were higher than the (124.76 ± 3.23)% observed for free QLNWD at the same concentration. Therefore, regardless of the preparation method employed, QLNWD-loaded NPs exhibited favorable biocompatibility.

### 2.7. Cell Uptake Capacity

To evaluate the targeting ability of pre-loading NPs and post-loading NPs toward activating RAW264.7 macrophages, cellular internalization of the NPs was investigated using laser scanning confocal microscopy (CLSM). FITC-labeled QLNWD NPs were prepared and incubated with RAW264.7 cells for 24 h to facilitate specific cellular uptake, followed by nuclear staining with DAPI. The free QLNWD group served as a control to assess the intracellular transport of the NPs. The 3D CLSM images (Figure 6B) depict that the green fluorescence signal from the NPs was significantly stronger than that observed in the free QLNWD group. Notably, some pre-loading NPs were found within the nucleus, whereas post-loading NPs were predominantly localized at the cell periphery. This difference may be attributed to the distinct behaviors of the two nanocarriers during cellular uptake. In addition, flow cytometry analysis (Figure 6C,D) demonstrated that the average fluorescence intensity of both pre-loading NPs and post-loading NPs was significantly higher compared to the free QLNWD group. This indicates that the NPs enhanced cellular uptake of QLNWD, a finding consistent with the CLSM observations. The results suggest that the NPs can ultimately be internalized by macrophages and exert their function.

## 3. Discussion

*Meretrix meretrix* is a marine organism with significant potential as a source of bioactive compounds [21]. A previous study has identified a pentapeptide, QLNWD, from its visceral hydrolysate, which exhibits immunomodulatory activity [4]. To enhance the applicability of QLNWD in oral drug delivery, we developed a novel and environmentally friendly nano-delivery system for its encapsulation. Our findings demonstrate that GBA NPs can effectively encapsulate QLNWD, and the choice of encapsulation method significantly influences the physicochemical properties of the resulting NPs. Specifically, although both types of nanoparticles were fabricated from the same raw materials, pre-loading NPs demonstrated higher encapsulation efficiency (87.74%), superior controlled release performance (37.8% at 12 h), and enhanced bioaccessibility (47.5%) of QLNWD. In contrast, post-loading NPs exhibited stronger co-localization with QLNWD (R = 0.95) and a smaller average particle size (94.27 nm). Both formulations effectively enhanced the cellular uptake of QLNWD by RAW264.7 macrophages. This study highlights the functional advantages of incorporating peptide drugs during nanoparticle formation by systematically comparing the behavioral differences of peptide molecules in the self-assembly process.

**Figure 7:**
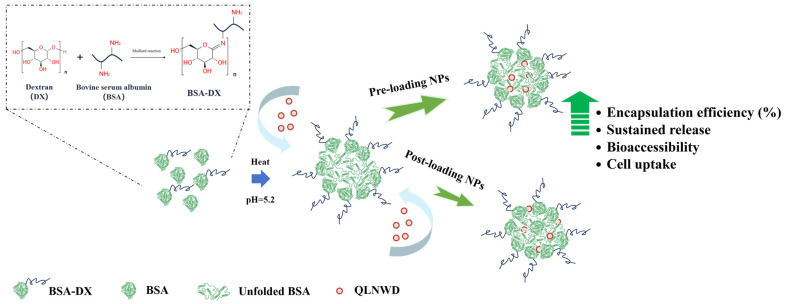
Synthesis scheme of the preparation of QLNWD-loaded GBA nanoparticles.

Bovine serum albumin, which lacks glycosylation, undergoes aggregation upon heating at its isoelectric point, a phenomenon attributable to the combined effects of charge shielding and exposure of hydrophobic interactions. This result is consistent with the findings of Fan et al. [22]. In this study, glycoprotein nanogel particles were prepared via thermal gelation of BSA-DX (grafting degree 21.90%) after dry-heating treatment for 9 h, and the formation mechanism is illustrated in Figure 7. At this stage, the uncrosslinked BSA underwent gelatinization upon heat treatment and constituted the core of the NPs. The BSA conjugated with dextran was localized in the shell of the NPs. The dextran chains extended outward from the nanoparticle shell, thereby inhibiting aggregation of the BSA core [15,23]. The absolute value of the zeta potential of GBA NPs is lower than that of pure BSA at pH 7, which indirectly indicates that BSA is embedded within the nanoparticle core, whereas the outer surface is shielded by conjugated dextran. The steric hindrance conferred by the polysaccharide chains enhances the colloidal stability of the system [20].

Interestingly, regardless of whether QLNWD is loaded before or after heating, its encapsulation significantly reduces the nanoparticle size. This observation contrasts with reports showing that encapsulation of most hydrophobic nutrients typically increases nanoparticle size [24], but similar trends have been observed during the encapsulation of selenium-containing peptides [25] and egg white peptides [26]. Since the peptide possesses self-assembly capability, QLNWD may function not only as a cargo molecule but also as an active participant in the assembly process. It is hypothesized that QLNWD can interact with biopolymer complexes (BSA-DX in GBA NPs) and serve as a more sophisticated and controllable functional template or inducer for self-assembly. This templating effect may promote the formation of a more compact nanostructure, providing a plausible explanation for the particle size reduction observed via DLS [27,28,29]. Our novel finding is that the particle size of post-loading NPs is significantly smaller than that of pre-loading NPs, which may be attributed to the diffusion-driven loading process of QLNWD into NPs in the post-loading method, thereby altering the properties of the particle-water interface [30]. Further supporting evidence is provided by the 2D fluorescence localization images, which show a high co-localization coefficient (R = 0.95) for post-loading NPs. This observation suggests that the post-loading method enables the formation of a uniform and dense peptide layer on the nanoparticle surface through electrostatic interactions, whereas the distribution of peptides in pre-loading NPs is less homogeneous [31]. However, the difference in R values alone cannot reflect variations in the penetration depth of QLNWD within the two types of nanoparticles. Therefore, in future studies, the intraparticle penetration behavior of peptide molecules should be evaluated using techniques such as z-stacked confocal imaging or fluorescence resonance energy transfer analysis.

A possible explanation for the lower encapsulation efficiency observed in the post-loading method is that the electrostatic and hydrophobic interactions between QLNWD and NPs constitute a dynamic equilibrium process. Previous studies on core–shell nanogels that encapsulate doxorubicin via diffusion have similarly demonstrated that increasing the pH of the mixed system enhances both hydrophobic interactions and electrostatic attractions between the drug and the NPs, thereby improving the doxorubicin loading rate [30]. The reduction in X-ray diffraction crystallinity following drug loading, together with the disappearance of the cold crystallization exothermic peak of QLNWD in DSC, confirmed that QLNWD was molecularly dispersed and stabilized in an amorphous state within the carrier matrix, with its physical mobility effectively restricted. This amorphous state contributes to the high encapsulation efficiency and controlled release properties of QLNWD, findings consistent with those reported in egg white peptide delivery systems [32,33]. Although the absolute value of the zeta potential of both nanoparticles was below 30 mV, their PDI remained below 0.4 after 28 days of storage at 4 °C, which can be attributed to a key factor: the cross-linking network formed by the heat-denatured gel, combined with the outer hydrophilic polysaccharide chains, provides substantial steric hindrance. This core–shell structure functions as a physical barrier in the physiological environment, effectively preventing nanoparticle aggregation [34,35].

The gastrointestinal digestion stability of glycosylated protein nanogels has also been confirmed in previous studies by Feng et al. [17]. This enhanced stability is attributed to the steric hindrance effect of the dextran corona and the reduction of protein amino groups during glycosylation, which decreases the proteolysis rate of nanogel particles and thereby prolongs the residence time of QLNWD within the NPs [36,37]. It was observed that the encapsulation of QLNWD into nanoparticles significantly enhanced its uptake by macrophages. This phenomenon may be attributed to the chemical properties of the GBA surface, such as the presence of extended glycans, which can be recognized by receptors on macrophage surfaces [38,39]. In the future, this potential mechanism should be further investigated through receptor blocking experiments to elucidate the underlying cellular transport pathways.

It is important to note that the cell uptake study did not pre-expose the NPs to gastrointestinal conditions [40]. While this experiment successfully demonstrates the macrophage-targeting potential of the NPs, future studies will include pre-treatment with SGF and SIF to better simulate the oral delivery pathway and quantify the residual uptake efficiency. In addition, a layer-by-layer self-assembly strategy could be employed to utilize the negative surface charge of existing nanoparticles for the electrostatic adsorption of a positively charged biocompatible polymer layer, such as chitosan or its derivatives (e.g., quaternized chitosan) [41,42]. This approach aims to significantly increase the absolute value of the zeta potential, thereby enhancing electrostatic repulsion and complementing the existing steric stabilization to construct a more robust and stable drug delivery system [43]. In summary, our study demonstrates that GBA NPs, particularly pre-loading NPs, exhibit promising potential as marine peptide-based nano-delivery systems by improving the controlled release and bioaccessibility of QLNWD at high EE (%).

## 4. Materials and Methods

### 4.1. Materials and Chemicals

The immunomodulatory peptide QLNWD and FITC-labeled QLNWD were synthesized from Shanghai Qiangyao Biotechnology Co., Ltd. (Shanghai, China), with purities of 98.46% and 98.56%, respectively. BSA (BSA amount ≥ 97%) and rhodamine B (RhB) were bought from Beijing Solarbio Science & Technology Co., Ltd. (Beijing, China). DX (molecular weight = 40 kDa) and DAPI staining solution were obtained from Shanghai Macklin Biochemical Co., Ltd. (Shanghai, China). Saliva, gastric fluid, and intestinal fluid were acquired from Shanghai Yuanye Bio-Technology Co., Ltd. (Shanghai, China).

The mouse macrophage RAW 264.7 cell line was purchased from the Type Culture Collection of the Chinese Academy of Sciences (Shanghai, China). RAW264.7 cell-specific culture medium and Dulbecco’s modified Eagle’s medium (DMEM) were supplied by Procell Life Science & Technology Co., Ltd. (Wuhan, Hubei, China). The Cell Counting Kit-8 was provided by White Shark Enterprise Management Consulting (Shenzhen) Co., Ltd. (Shenzhen, Guangdong, China). Phosphate-buffer solution (PBS) (10×) was obtained from Gibco, Thermo Fisher Scientific (Grand Island, NY, USA).

### 4.2. Synthesis of BSA-DX Conjugates

The synthesis of BSA-DX conjugates was carried out via a dry Maillard reaction according to a previously described method [22]. BSA (1%, *w*/*v*) and DX (2%, *w*/*v*) were dissolved in ultrapure water and allowed to hydrate fully over 12 h. Subsequently, the pH of the solution was adjusted to 7.0. The solution was freeze-dried, and the resulting powder was transferred to a constant temperature and humidity chamber (LJ-70B, Guangdong Lijia Industrial Co., Ltd., Dongguan, China) and incubated at 60 °C under 79% relative humidity, maintained with a saturated KBr solution, for varying durations (0, 3, 6, 9, 12, and 24 h) to obtain BSA-DX conjugates. After the conjugation process, all samples were collected and stored at –20 °C pending further analysis.

### 4.3. Characterization of BSA-DX Conjugates

#### 4.3.1. DG and Browning Index Measurement

The DG of the BSA-DX conjugate was assessed using the *o*-phthalaldehyde method, based on the measurement of reduced free amino groups [44]. The Browning Index allows for quantitative analysis of the BSA-DX glycosylation process [45]. The absorbance of the conjugate aqueous solution was measured at 420 nm and 294 nm using an ultraviolet spectrophotometer (UV-1900i, Shimadzu Corporation, Kyoto, Japan) to assess the extent of final browning and the occurrence of intermediate Maillard reaction.

#### 4.3.2. SDS-PAGE

BSA and BSA-DX conjugates (protein concentration 5 mg/mL) were heated in SDS loading buffer (40 mM Tris-HCl, pH 6.8, 200 mM DTT, 4% SDS, 0.03% bromophenol blue, 40% glycerol) for 5 min and subsequently separated via electrophoresis on a 4–20% gradient gel (10 μL). Electrophoresis was performed under a constant voltage of 100 V. Post-electrophoresis, the gel was dyed with Coomassie Brilliant Blue G-250 [46] and scanned using a gel imaging system (GelDocTM XR+, Bio-Rad Laboratories, Hercules, CA, USA).

#### 4.3.3. FT-IR

Spectral analysis of all samples was performed on a spectrometer (TENSOR27, BRUKER OPTICS GmbH, Ettlingen, Germany). Measurements were carried out in the range of 4000–400 cm^−1^ with a spectral resolution of 4 cm^−1^, accumulating 32 scans per spectrum. A background spectrum collected with KBr was subtracted before each sample measurement.

#### 4.3.4. Fluorescence Spectroscopy

The fluorescence spectrum was measured for the sample solution (0.1 mg/mL) on a fluorescence spectrophotometer (RF-5301, Shimadzu Corporation, Kyoto, Japan). The measurement was performed with an excitation wavelength of 280 nm, and the emission was recorded between 290 and 450 nm using a constant slit width of 5 nm for both monochromators.

### 4.4. Preparation of Nanoparticle

For the preparation of GBA NPs, BSA-DX conjugates (10 mg/mL) (0, 3, 6, 9, 12, and 24 h) were dispersed in ultrapure water and stirred to ensure complete hydration. The pH was adjusted to 5.2 with 0.1 M HCl. The solution was heated at 80 °C for 30 min, rapidly cooled to room temperature using an ice bath, and then stored at 4 °C or freeze-dried.

### 4.5. Preparation of QLNWD-Loaded GBA NPs

**Figure 8:**
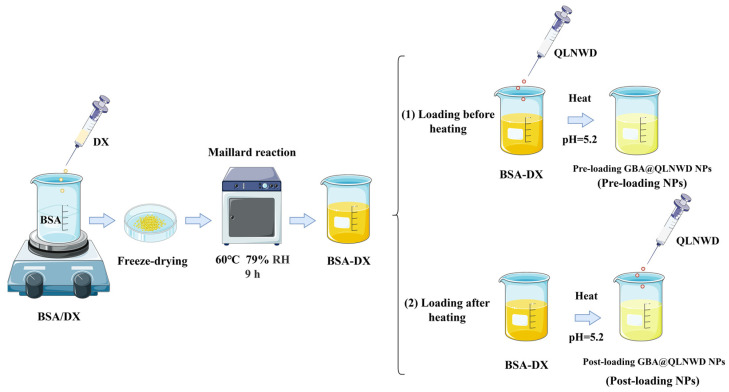
Fabrication of QLNWD-loaded GBA nanoparticles with different adding sequences.

Two distinct loading strategies, pre-loading and post-loading, were adopted to prepare GBA NPs encapsulating QLNWD. These strategies differed based on the addition of QLNWD before or following nanogel formation, and the preparation protocol is shown in Figure 8.

For the pre-loading approach, a 10 mg/mL QLNWD solution was prepared and mixed with a 10 mg/mL solution of the BSA-DX conjugate (9 h) (volume ratio of QLNWD: BSA-DX = 1:9). After being magnetically stirred at room temperature for 2 h, the mixture had its pH adjusted to 5.2 with 0.1 M HCl. The solution was subsequently heated at 80 °C for 30 min and finally cooled rapidly in an ice bath. The resulting NPs were labeled as pre-loading NPs.

For the post-loading approach, the BSA-DX conjugate solution (10 mg/mL) was fully hydrated, and the pH was adjusted to 5.2 with 0.1 M HCl. The solution was subsequently heated at 80 °C for 30 min, rapidly cooled to room temperature using an ice bath, and mixed with a 10 mg/mL QLNWD solution (volume ratio of QLNWD:BSA-DX = 1:9). Following adjustment of the final pH to 7.0, the nanoparticle was magnetically stirred at room temperature for 6 h. The resulting NPs were labeled as post-loading NPs.

Meanwhile, free QLNWD aqueous solution (1 mg/mL) and GBA NPs without QLNWD were prepared using the same protocol for use as controls. The resulting samples were stored at 4 °C or freeze-dried for subsequent analysis.

### 4.6. EE of QLNWD

Fresh NPs were centrifuged at 4 °C (10,000 rpm, 10 min) to remove larger aggregates. To isolate unencapsulated QLNWD, the supernatant was centrifuged at 8000 rpm for 20 min in an ultrafiltration tube (molecular weight cutoff of 1 kDa). The concentration of QLNWD in the permeate was quantified via HPLC (Arc, Waters Corporation, Milford, MA, USA) using a standard curve. The EE of the NPs was calculated as follows:

(1) $$EE(\%)=(1-\frac{free\;QLNWD\;in\;the\;supernatant}{total\;QLNWD\;content})\times 100$$

### 4.7. Characterization of NPs

#### 4.7.1. Determination of Particle Size, Zeta Potential, and Turbidity

The average particle size, PDI, and zeta potential of the NPs were measured by a Marvin laser granulometer (ZETASIZER NANO ZSE, Malvern Instrument Co., Ltd., Worcestershire, UK). Sample turbidity was evaluated by measuring transmittance at 600 nm using a ultraviolet spectrophotometer. The pH was adjusted as needed with 0.1 M NaOH or HCl. The refractive indices of water and NPs were 1.33 and 1.45, respectively. All measurements were performed at 25 °C with three replicates per sample.

#### 4.7.2. Morphological Characterization

The morphology of the QLNWD-loaded nanoparticle aqueous solution was characterized using transmission electron microscopy (HT7800, HITACHI High-Tech Corporation, Tokyo, Japan) according to the method described by Yang et al. [26].

FITC-labeled QLNWD and Rho-stained GBA NPs were utilized to prepare NPs using two distinct loading methods under dark conditions. The microstructure of the samples was examined using CLSM (STELLARIS 5, Leica Microsystems GmbH, Wetzlar, Germany). The Pearson correlation coefficient was computed using Fiji (distribution of ImageJ, version 1.54p).

#### 4.7.3. FT-IR and XRD

QLNWD, GBA NPs, pre-loading NPs, and post-loading NPs were analyzed by a TENSOR27 spectrometer with 32 scans across the wavenumber range of 4000–400 cm^−1^ at a resolution of 4 cm^−1^. Prior to sample measurement, the KBr background spectrum was acquired and subtracted to ensure spectral accuracy. The XRD data of the powders were obtained using an X-ray diffractometer (Ultima IV, Rigaku Corporation, Tokyo, Japan). The instrument was configured with a 1° divergence slit and a 0.1 mm receiving slit. Data were acquired in the 2θ range of 5–60° with a scanning speed of 2° per minute.

#### 4.7.4. DSC

The thermal properties of samples were characterized using a differential scanning calorimeter (Jupiter STA 449 F3, NETZSCH-Gerätebau GmbH, Selb, Germany). Powdered samples (around 7 mg) were sealed in hermetic aluminum pans and heated from 30 to 250 °C at a rate of 10 °C/min under a nitrogen purge gas flow of 30 mL/min [47].

### 4.8. Stability of the Delivery System

#### 4.8.1. Storage Stability

After dispersing 100 mg of nanoparticle powder in 10 mL of ultrapure water, the solution was stored at 4 °C for 28 days. The particle size and PDI of the samples were analyzed with a Marvin laser granulometer.

#### 4.8.2. pH Stability

The nanoparticle solution was mixed with an equal volume of either 0.1 mM HCl or 0.1 mM NaOH, and the pH was adjusted to a range of 2.0 to 7.0. Following a 1-h incubation period at room temperature, the particle size and PDI of the sample were measured [48].

#### 4.8.3. In Vitro Release Study of QLNWD in NPs

The release profile of QLNWD in NPs was evaluated according to a published dialysis method [49]. A 2 mL aliquot of QLNWD (1 mg/mL) nanoparticle solution was introduced into a presoaked dialysis bag (molecular weight cutoff of 3500 Da). The bag was immersed in 20 mL of simulated gastric fluid (SGF, enzyme-free), which had been preheated at 37 °C for 30 min and adjusted to pH 2. The entire system was incubated at 37 °C under continuous agitation at 120 rpm. After 2 h, the pH was adjusted to 7.0, and 20 mL of simulated intestinal fluid (SIF, enzyme-free), also preheated at 37 °C for 30 min and adjusted to pH 7.0, was added to the system. At predetermined intervals, 1 mL of the release medium was sampled and immediately replenished with an equal volume of fresh SGF/SIF. Samples without QLNWD served as the blank control, and the cumulative release was determined as follows:

(2) $$Cumulative\;release\;(\%)=\frac{\left(V_{0}C_{n}+V_{e}\sum_{1}^{n-1}C_{n-1}\right)}{total\;QLNWD\;content}\times 100$$

where *V_0_* refers to the total volume of simulated gastric or intestinal fluid, *C_n_* refers to the concentration of QLNWD at the nth time, and *V_e_* refers to the volume of each sample.

#### 4.8.4. Bioaccessibility Evaluation of QLNWD

The bioaccessibility of QLNWD-loaded NPs was assessed based on a previously published method [26]. Briefly, 5 mL of the sample solution was mixed with 5 mL of simulated saliva and vortexed for 5 min to simulate the oral digestion phase. The pH of the mixture was then adjusted to 2.0, followed by the addition of 20 mL SGF (final pepsin activity = 2000 U/mL), to simulate gastric digestion at 37 °C under continuous shaking (120 rpm) for 2 h. Subsequently, the pH was set to 7.0, and 20 mL SIF (containing 100 U/mL pancreatin in the final digestive medium) was added to mimic intestinal digestion, which was also carried out at 37 °C with shaking at 120 rpm for 2 h. Finally, the digested sample underwent a 10-min heating in a boiling water bath, followed by cooling to room temperature and centrifugation at 10,000 rpm at 4 °C for 10 min. The supernatant was collected and filtered through a 0.22 μm membrane, and the bioaccessibility of QLNWD was subsequently calculated using Equation (3).

(3) $$Bioaccessibility(\%)=\frac{QLNWD\;concentration\;in\;the\;mixed\;micelle\;phase}{QLNWD\;concentration\;in\;total\;digesta}\times 100$$

### 4.9. Cell Viability

The effects of free QLNWD, GBA NPs, pre-loaded NPs, and post-loaded NPs on the viability of RAW264.7 macrophages were evaluated by employing the CCK-8 kit following the method of Z. Li et al. [50]. In brief, RAW264.7 cells (1.0 × 10^5^ cells/mL) were seeded into 96-well plates and incubated for 24 h at 37 °C under 5% CO_2_. Thereafter, the culture medium was substituted with media containing the respective samples. GBA NPs at concentrations of 0.5, 1, 2, 4, and 8 mg/mL. The final concentrations of QLNWD in free QLNWD, pre-loaded NPs, and post-loaded NPs were set at 50, 100, 200, 400, and 800 μg/mL, respectively. The blank control group received DMEM culture medium without any treatment. After an additional 24 h of incubation, the culture medium was removed, and 10 μL of CCK-8 reagent was added to each well. The cells were then incubated in the cell incubator for 1 h. The absorbance at 450 nm was measured using a microplate reader (Varioskan Flash, Thermo Fisher Scientific, Waltham, MA, USA), and the relative cell viability was calculated as follows:

(4) $$Cell\;viability\;(\%)=\frac{A}{A_{0}}\times 100$$

where *A*_0_ refers to the control absorbance and *A* refers to the absorbance value of the sample.

### 4.10. Cell Uptake

The cellular uptake of QLNWD by RAW264.7 macrophages was assessed using CLSM [51]. Firstly, RAW264.7 macrophages were cultured in a specialized medium in an incubator maintained at 37 °C with 5% CO_2_. The cells were seeded into 24-well plates at a density of 1.0 × 10^5^ cells/mL and cultured under standard conditions (37 °C, 5% CO_2_) for 24 h. Subsequently, NPs loaded with FITC-labeled QLNWD were introduced, with free FITC-labeled QLNWD serving as the control. After 24 h of incubation, the culture medium was removed, and the cells were gently washed three times with PBS. Next, the cells were fixed with 4% paraformaldehyde for 20 min at room temperature, followed by another PBS wash. Nuclei were stained with DAPI in the dark for 5 min and visualized using CLSM.

The cellular uptake of QLNWD in RAW264.7 cells was quantified by flow cytometry [52]. Briefly, RAW264.7 cells (1.0 × 10^5^ cells/mL) were co-cultured with either free FITC-labeled QLNWD or FITC-labeled QLNWD-loaded NPs in 24-well plates and incubated at 37 °C for 24 h in an incubator maintained at 5% CO_2_. Following incubation, the cells were rinsed three times with PBS and then digested with trypsin. The cell solution was centrifuged at 1500 rpm/min for 5 min to collect cells. After removal of the supernatant, the cells were resuspended in PBS buffer and immediately analyzed by a flow cytometer (CytoFLEX S, Beckman Coulter, Inc., Brea, CA, USA). Data analysis was performed using FlowJo software (version 10.9.0).

### 4.11. Statistical Analysis

All data are expressed as mean ± standard deviation (SD). Differences between groups were evaluated by one-way analysis of variance (ANOVA) with SPSS version 27 (IBM, Armonk, NY, USA), with post hoc comparisons performed using the least significant difference (LSD) test. A *p*-value of less than 0.05 was considered statistically significant.

## 5. Conclusions

In summary, GBA NPs were prepared via isoelectric point heating-induced self-assembly at pH 5.2. QLNWD was loaded into NPs using two methods: pre-loading and post-loading. The loading of QLNWD leads to a reduction in the average particle size of NPs, and both the EE and co-localization efficiency of QLNWD vary depending on the order of its addition. Regardless of the preparation method, NPs loaded with QLNWD predominantly exhibited a core–shell structure facilitated by hydrogen bonding, hydrophobic interactions, and electrostatic forces. Additionally, both types of NPs exhibited good biocompatibility and stability and enhanced the bioavailability of QLNWD during in vitro simulated gastrointestinal digestion. Compared to free QLNWD, nanoparticle encapsulation significantly increased macrophage uptake of the peptide. Although our study has demonstrated the advantages of peptide drugs participating in the nanoparticle formation process, further research is required to investigate the self-assembly kinetics of peptides. In addition, the cellular transport and uptake of nanoparticles, their in vivo efficacy, and cell targeting capability require systematic animal studies in subsequent work for thorough validation and optimization.

## Figures and Tables

**Table 1:** The particle size, polydispersity index (PDI), and zeta potential of glycoprotein nanoparticles (GBA NPs), pre-loading NPs, and post-loading NPs.

Sample	Particle Size (nm)	PDI	Zeta Potential (mV)
GBA NPs	110.84 ± 0.71 ^a^	0.223 ± 0.011 ^b^	−7.38 ± 0.57 ^b^
Pre-loading NPs	105.51 ± 0.46 ^b^	0.254 ± 0.010 ^a^	−8.42 ± 0.27 ^a^
Post-loading NPs	94.27 ± 0.27 ^c^	0.226 ± 0.002 ^b^	−8.12 ± 0.12 ^ab^

Note: Values are mean ± standard deviation of three replicates. Different letters indicated the significant difference at *p* < 0.05.

## Data Availability

Data are available upon request.

## Abbreviations

The following abbreviations are used in this manuscript:

BSA	Bovine serum albumin
CCK-8	Cell Counting Kit-8
CLSM	Confocal laser scanning microscope
DG	Degree of glycosylation
DLS	Dynamic light scattering
DX	Dextran
EE	Encapsulation efficiency
FT-IR	Fourier transform-infrared spectroscopy
GBA NPs	Glycosylated BSA nanoparticles
PBS	Phosphate-buffer solution
PDI	Polydispersity index
QLNWD	Gln-Leu-Asn-Trp-Asp
R	Pearson’s correlation coefficient
RhB	Rhodamine B
SDS-PAGE	Sodium dodecyl sulfate-polyacrylamide gel electrophoresis
SGF	Simulated gastric fluid
SIF	Simulated intestinal fluid
TEM	Transmission electron microscopy
Tm	Melting temperature
XRD	X-ray diffraction
